# Impact of Junction Length on Supercurrent Resilience against Magnetic Field in InSb-Al Nanowire Josephson Junctions

**DOI:** 10.1021/acs.nanolett.2c04485

**Published:** 2023-05-22

**Authors:** Vukan Levajac, Grzegorz P. Mazur, Nick van Loo, Francesco Borsoi, Ghada Badawy, Sasa Gazibegovic, Erik P. A. M. Bakkers, Sebastian Heedt, Leo P. Kouwenhoven, Ji-Yin Wang

**Affiliations:** † QuTech and Kavli Institute of Nanoscience, Delft University of Technology, 2600 GA Delft, The Netherlands; ‡ Department of Applied Physics, Eindhoven University of Technology, 5600 MB Eindhoven, The Netherlands

**Keywords:** Josephson junction, resilient supercurrent, superconducting interference

## Abstract

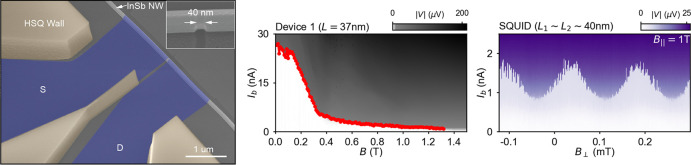

Semiconducting nanowire Josephson junctions represent an attractive platform to investigate the anomalous Josephson effect and detect topological superconductivity. However, an external magnetic field generally suppresses the supercurrent through hybrid nanowire junctions and significantly limits the field range in which the supercurrent phenomena can be studied. In this work, we investigate the impact of the length of InSb-Al nanowire Josephson junctions on the supercurrent resilience against magnetic fields. We find that the critical parallel field of the supercurrent can be considerably enhanced by reducing the junction length. Particularly, in 30 nm long junctions supercurrent can persist up to 1.3 T parallel field—approaching the critical field of the superconducting film. Furthermore, we embed such short junctions into a superconducting loop and obtain the supercurrent interference at a parallel field of 1 T. Our findings are highly relevant for multiple experiments on hybrid nanowires requiring a magnetic-field-resilient supercurrent.

Semiconducting nanowire Josephson junctions (JJs) are widely used as a versatile platform for studying various physical phenomena that arise in semiconductor–superconductor hybrid systems. Therein, the III–V semiconductors have attracted a particular interest in exploring the anomalous Josephson effect,^1−4^ topological superconductivity^5−11^ and the Josephson diode effect,^12−14^ due to their strong spin–orbit interaction and large *g* factor. Recently, the Josephson diode effect has been exceptionally intriguing in both theory^15−18^ and experiment.^13,14,19−22^ In the above research works, an indispensible ingredient is the breaking of time reversal symmetry, which is normally achieved via external magnetic fields. However, an external magnetic field generally suppresses the supercurrent through a hybrid nanowire JJ—therefore significantly limiting the parameter space for addressing the aforementioned effects in hybrid nanowires. Preserving the supercurrent in hybrid nanowire JJs at high magnetic fields thus becomes critically important. Selecting high critical field superconductors, such as NbTiN,^23^ Pb,^24^ Sn,^25^ or Al doped by Pt,^26^ seems to be an option for improving the magnetic field compatibility of the supercurrent. However, none of these material platforms have yielded a supercurrent at high magnetic fields. Moreover, it has been observed that the supercurrent of nanowire JJs generally vanishes at magnetic fields far below the critical field of the superconducting film.^27,28^ Searching for an alternative way to improve the supercurrent resilience against magnetic field in nanowire JJs is thus needed. In spite of extensive works on nanowire JJs with either evaporated superconducting contacts^28−31^ or epitaxially grown superconducting shells,^27,32,33^ a potential impact of the junction length on supercurrent performance in magnetic fields has not been systematically investigated.

In this work, we have studied InSb-Al nanowire JJs with the junction length *L* varying from 27 to 160 nm. The junction length has been found to be an essential parameter that determines the supercurrent evolution in a parallel magnetic field. In the long devices (*L* ≈ 160 nm), the supercurrent is suppressed quickly in a magnetic field and fully vanishes at parallel fields of ∼0.7 T. In contrast, the supercurrent in short devices (*L* ≈ 30 nm) persists up to parallel fields of ∼1.3 T, approaching the critical in-plane magnetic field of the Al film (∼1.5 T^26,27,34^). Despite the influence of the electrochemical potential in the juntions, the resilient supercurrent is present only in the short devices (*L* ≈ 30 nm). We exploit this property to realize a magnetic-field-resilient superconducting quantum interference device (SQUID). At a magnetic field of 1 T, the supercurrent through the device displays the characteristic oscillatory pattern as a function of the magnetic flux through the loop. We expect that our demonstration of magnetic-field-resilient supercurrent in remarkably short nanowire JJs offers a new approach to improving the field compatibility of not only SQUIDs but many other hybrid nanowire devices utilizing the Josephson effect at high magnetic field.

The hybrid nanowire JJs are fabricated by recently developed shadow-wall deposition techniques.^27,34^ As shown in Figure 1a, a scanning electron microscope (SEM) image of a representative InSb-Al nanowire JJ device is taken at a tilted angle and shown in false colors. Source (S) and drain (D) superconducting leads (blue) are formed via an in situ angle deposition of Al film after the preparation of a clean and oxide-free InSb nanowire^35^ interface (see the Methods section in the Supporting Information). Prepatterned dielectric shadow walls (yellow) selectively define the nanowire sections that are exposed to the Al flux during the deposition. The junction length is determined by the width of the shadow wall in the vicinity of the nanowire. In comparison with the etched dielectric shadow walls used in recent works^27,33,34^ or previous evaporation-defined JJs,^28−31^ here we use lithographically defined shadow walls whose dimensions therefore can be as small as 20 nm. This allows us to precisely control the length of nanowire JJs and to achieve surpassingly short junctions, as shown in the inset SEM image in Figure 1a. In this work, we present nine nanowire JJ devices (Devices 1–9) with the junction length *L* in the range of 27–160 nm and one InSb-Al nanowire SQUID with two junctions of ∼40 nm. The diameter of the nanowires is ∼100 nm. An overview of nine nanowire JJ devices is shown in Figure S2 in the Supporting Information.

**Figure 1:**
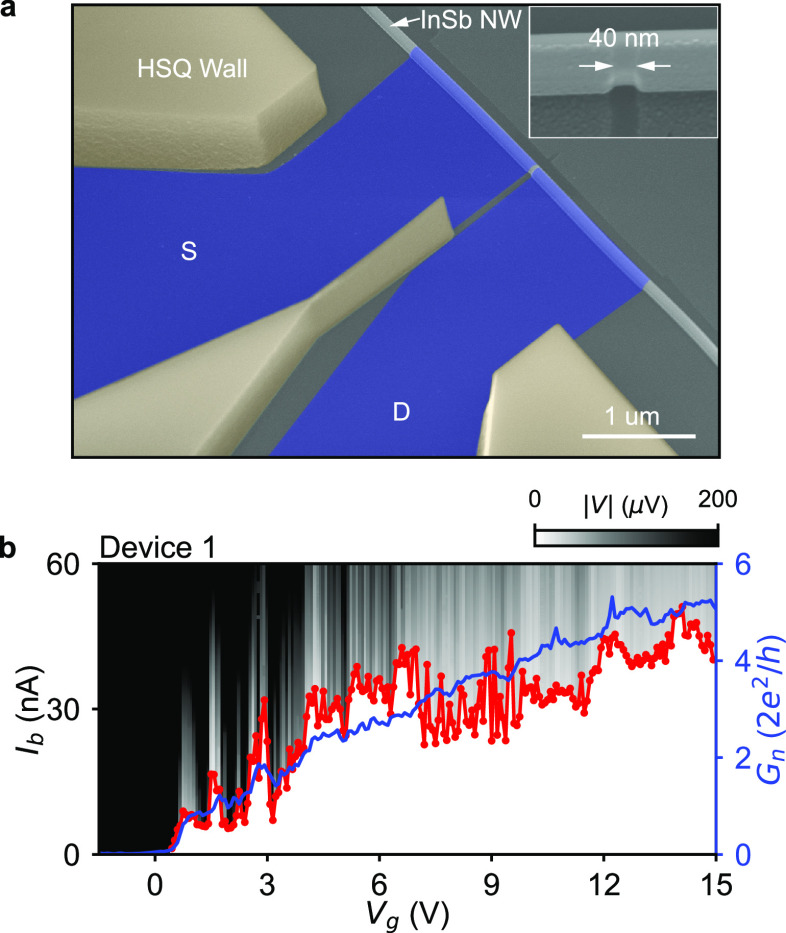
Basic characterization of a nanowire Josephson junction device. (a) False-colored SEM image depicting a representative JJ device with a semiconducting InSb junction defined between the source (S) and drain (D) superconducting Al leads (blue). The junction length is determined by the hydrogen silsesquioxane (HSQ) (yellow) shadow-wall structure. An enlargement at the junction is shown in the inset. The back side of the substrate is used as a global back gate. (b) Zero-field dependence of switching current *I*_sw_ (red) and normal state conductance *G*_n_ (blue) on the back gate voltage *V*_g_, overlapped onto the *I*_b_–*V*_g_ two-dimensional (2D) map taken for Device 1 (with junction length *L* = 37 nm).

Electrical transport measurements on the nanowire Josephson junction devices have been performed at ∼20 mK in a dilution refrigerator equipped with a vector magnet. A four-terminal setup is used for dc-current bias *I*_b_ measurements. Conductance measurements have employed a two-terminal setup with a dc-voltage bias *V*_b_ and a 10 μV ac excitation (see more details in the Supporting Information). The back side of the substrate is used as a back gate, and an applied voltage *V*_g_ acts globally on the entire nanowire. Figure 1b shows how the switching current *I*_sw_ (red) and the normal state conductance *G*_n_ (blue) depend on *V*_g_ at zero magnetic field for Device 1. The switching current *I*_sw_ is extracted from the (*V*, *I*_b_) traces (see the Data analysis section in the Supporting Information). The normal state conductance *G*_n_ is obtained in the voltage-bias range 1 mV < |*V*_b_| < 2 mV—well above the double value of the induced superconducting gap of the leads (2Δ ≈ 0.5 meV). The conductance measurements from which *G*_n_ and Δ are extracted are shown in Figures S3 and S9. By increasing *V*_g_, both *I*_sw_ and *G*_n_, in spite of fluctuations, become larger as the carrier states in the junction get populated and more subbands contribute to transport. At *V*_g_ = 15 V, *G*_n_ and *I*_sw_ reach up to ∼5*G*_0_ (*G*_0_ = 2*e*^2^/*h*) and ∼50 nA, respectively. The remaining nanowire JJs (Devices 2–9) show comparable zero-field properties, as shown in Figures S3 and S4. The high tunability of *G*_n_ as well as of *I*_sw_ enables the systematic investigation of the junctions in different electrochemical potential regimes.

Hybrid nanowire JJs have been shown to exhibit a supercurrent evolution in a parallel magnetic field *B* that is strongly affected by the electrochemical potential of the semiconducting junction.^28^ Therefore, when exploring the resilience of switching current in a parallel *B* field, the electrochemical potential of a junction has to be taken into account. In the following, the switching current dependence on *V*_g_ and the parallel *B* field is studied for two JJs of significantly different lengths. In Figure 2a,b, we show how the switching current *I*_sw_ evolves with *V*_g_ and *B* for Device 2 (*L* = 31 nm) and Device 7 (*L* = 157 nm), respectively. *I*_sw_ is extracted from the corresponding (*V*, *I*_b_) traces taken at each setting of *V*_g_ and *B*. As shown in Figure 2a, the short device shows a remarkable supercurrent resilience with the supercurrent persisting above a parallel field of 1 T. A linecut at 1 T (red bar) is taken, and the corresponding data are shown in Figure 2c. *I*_sw_ (red trace) continuously persists over an ∼3.5 V interval of *V*_g_. As a comparison, *I*_sw_ drops more rapidly with magnetic field in the long device, as shown in Figure 2b. Figure 2d shows that at 0.6 T the supercurrent is barely detectable. Besides this apparent difference, the switching current behaviors in Figure 2a,b still show some similarities. Namely, *I*_sw_ of both devices manifests a better resilience against the magnetic field in an intermediate gate interval between the pinch-off and the fully open regime—(−0.5, 3) V interval for the short device and (4, 10) V interval for the long device (see Figure S5). The switching current ubiquitously fluctuates in the intermediate gate intervals. We suspect that both few-mode interference^28^ and finite contact barriers^29^ may lead to such fluctuations in supercurrent as well as in normal conductance. For a gate voltage above these intervals *I*_sw_ in both devices vanishes more rapidly in the magnetic field, especially at *B* > 0.3 T. The suppression of supercurrent in at large positive *V*_g_ or high magnetic fields could be explained by a destructive interference between multiple modes.^1,2,28^ Another explanation could be a gate-tuned semiconductor–superconductor hybridization,^36,37^ which is addressed in the discussion part following Figure 4. A ubiquitous feature in Figure 2a,b is that, as the magnetic field is increased, certain intervals in the intermediate gate regime support more resilient supercurrent. In these *V*_g_ intervals we define the “resilient gate settings *V*_g,res_” (blue markers in Figure 2c,d). In this work, we quantify the impact of junction length on supercurrent resilience against magnetic field in two ways. The first way is to compare the supercurrent critical fields of different junctions at their *V*_g,res_, which is addressed in Figure 3. The second way is to compare the supercurrent averaged over a gate range at a finite magnetic field, which is shown in Figure 4.

**Figure 2:**
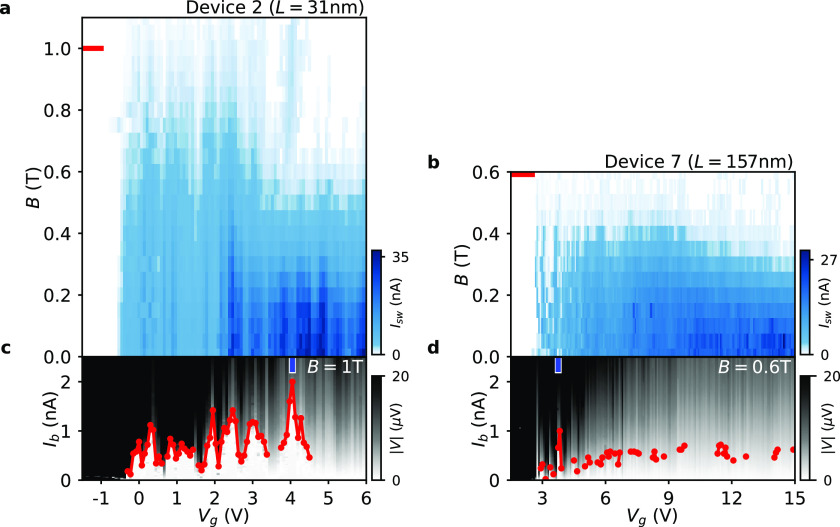
Dependence of switching current on the gate voltage and parallel magnetic field for (a) Device 2 (*L* = 31 nm) and (b) Device 7 (*L* = 157 nm). Each data point in the *V*_g_–*B* 2D map in (a) and (b) is extracted from the corresponding (*I*_b_, *V*) trace as the gate voltage *V*_g_ and the parallel magnetic field *B* are swept. The red markers in (a) and (b) correspond to the magnetic fields *B* = 1 T and *B* = 0.6 T at which the *I*_b_–*V*_g_ 2D maps in (c) and (d) are shown, respectively. In these maps the red traces correspond to the extracted switching current *I*_sw_. More analogous 2D maps at lower fields are displayed in Figure S5 in the Supporting Information. The blue markers in (c) and (d) denote the gate settings with enhanced supercurrent.

**Figure 3:**
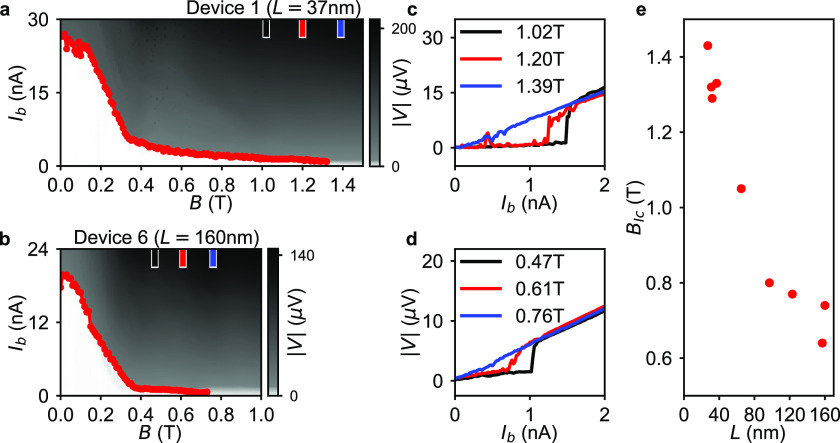
Critical parallel magnetic field of switching current. Dependence of the switching current (red) on *B* at the resilient gate settings *V*_g,res_ for (a) Device 1 (*L* = 37 nm) and (b) Device 6 (*L* = 160 nm). In each 2D map the extracted switching current *I*_sw_ up to the critical parallel field is plotted in red. The critical parallel fields of the switching current in (a) and (b) are *B*_Ic_ = 1.33 T and *B*_Ic_ = 0.74 T, respectively. Black, red, and blue markers in (a) and (b) have the corresponding linecuts shown in (c) and (d). In (e) the dependence of the critical parallel field *B*_Ic_ is plotted for Devices 1–9 versus the junction length *L*. Note that the uncertainty of *B*_Ic_ is not added in the plot and the amount is within 20 mT for all data points.

**Figure 4:**
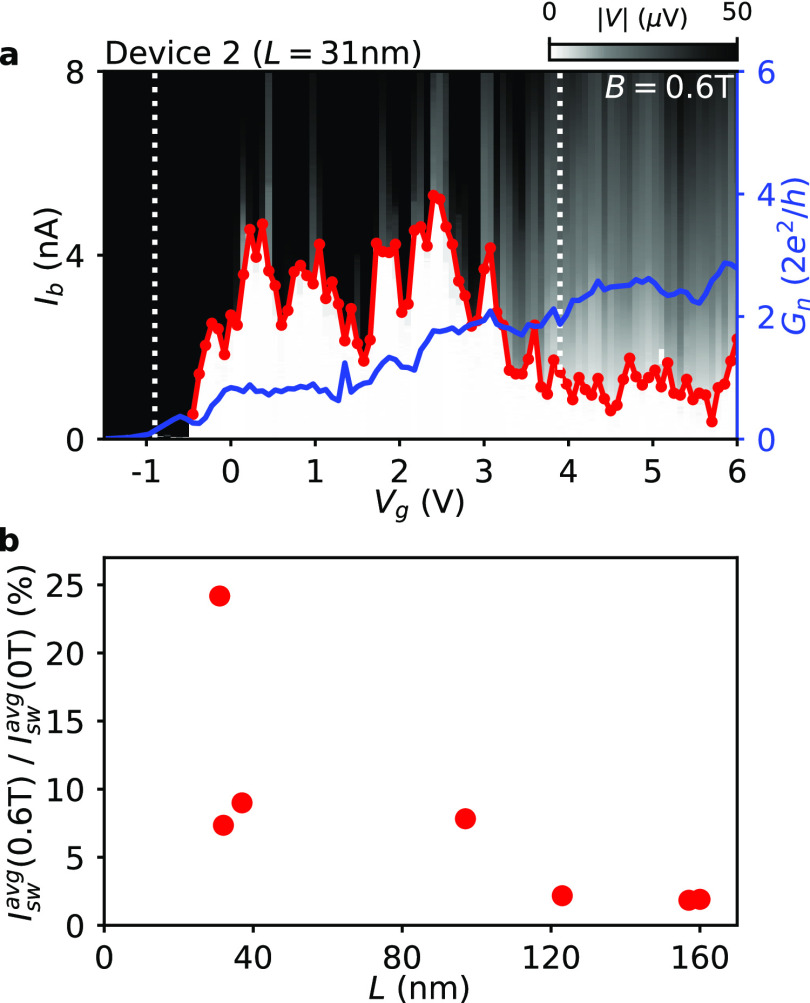
Resilience of switching current in the junctions tunability ranges. (a) Dependence of the switching current *I*_sw_ (red) on the gate voltage *V*_g_ at the parallel magnetic field *B* = 0.6 T for Device 2 (*L* = 31 nm). Two white vertical lines mark the gate interval over which the normal state conductance *G*_n_ of the device (blue) is tuned from 0.1*G*_0_ to 2*G*_0_. In this gate range the switching current is averaged and the *I*_sw_^avg^(0.6 T) value is obtained. Analogously, from the switching current dependence on *V*_g_ at zero field the average value *I*_sw_^avg^(0 T) is calculated. (b) Dependence of the ratio *I*_sw_^avg^(0.6 T)/*I*_sw_^avg^(0 T) on the junction length *L* for Devices 1–7.

In Figure 3 we focus on the supercurrent at the resilient gate settings *V*_g,res_. For Devices 1–7 we determine the *V*_g,res_ values as described in Figure S6, while for Devices 8 and 9 we choose *V*_g_ = 15 V. The normal conductance *G*_n_ at *V*_g,res_ is normally of a few *G*_0_ (*G*_0_ = 2*e*^2^/*h*), corresponding to a few transport modes, and the value does not show an obvious dependence on the junction length. Figure 3a shows the voltage drop *V* over the junction as a function of *I*_b_ and the parallel magnetic field *B* for Device 1 (*L* = 37 nm). The red dotted line marks the extracted switching current *I*_sw_ at different *B* fields. Three linecuts (black, red, and blue) are shown in Figure 3c—demonstrating more than 1 nA supercurrent at the parallel field of 1.2 T. Figure 3b,d shows the results for Device 6 (*L* = 160 nm) obtained at its *V*_g,*res*_ setting. From the overlaid red trace it can be seen that the supercurrent vanishes at ∼0.75 T, as confirmed by the linecuts shown in Figure 3d. Analogous measurements of the switching current evolution with parallel field are carried out for all nine devices (see Figure S7 in the Supporting Information). Finally, these *I*_sw_(*B*) dependences allow for the extraction of the maximal critical parallel magnetic field of switching current *B*_Ic_ for each Device 1–9. By plotting *B*_Ic_ versus the junction length *L* in Figure 3e, it can be seen how the junction length influences the measured critical field of the supercurrent. We reproducibly reach the critical fields of ∼1.3 T in the sub-40 nm junctions while *B*_Ic_ drops gradually to ∼0.7 T in the longest junctions.

As a next step, we evaluate the supercurrent resilience over a broader gate interval. As our nanowire JJs are highly tunable, in Figure 4 their supercurrent resilience against the parallel magnetic field is studied over the gate ranges in which the junctions are in the few-mode regimes. Figure 4a shows the voltage drop *V* as a function of *I*_b_ and *V*_g_ at a parallel field of 0.6 T for Device 2 (*L* = 31 nm), together with *I*_sw_ (red trace) and the normal state conductance *G*_n_ (blue trace). To quantify the supercurrent resilience, the switching current in Figure 4a is averaged in the *V*_g_ range corresponding to 0.1*G*_0_ < *G*_n_(*V*_g_) < 2*G*_0_ (denoted by the two white dotted lines) and the obtained average switching current is *I*_sw_^avg^(0.6 T) = 2.73 nA. Such a moderate gate range is selected to keep enough supercurrent flow and meanwhile diminish the multiple mode inteference effects. An analogous averaging is done for the *I*_sw_(*V*_g_) dependence measured at zero field, and the obtained average switching current at zero field is *I*_sw_^avg^(0 T) = 11.29 nA (see Figure S4 for the zero-field dependence and the average value). By calculating the ratio *I*_sw_^avg^(0.6 T)/*I*_sw_^avg^(0 T), it can be inferred that the junction of Device 2 preserves on average ∼25% of its zero field switching current when the parallel field of 0.6 T is applied. The identical procedures of calculating the average switching currents and the *I*_sw_^avg^(0.6 T)/*I*_sw_^avg^(0 T) ratios are carried out for Device 1–7 (see Figure S4 and Figure S8 in the Supporting Information). The dependence of the *I*_*sw*_^*avg*^(0.6 T)/*I*_*sw*_^*avg*^(0 T) on the junction length *L* is shown as red dots in Figure 4b. It can be noticed that at finite parallel field the shorter junctions preserve larger fractions of the corresponding zero-field supercurrent in the described conductance ranges. The ratio *I*_sw_^avg^(0.6 T)/*I*_sw_^avg^(0 T) drops rapidly around *L* ≈ 100 nm, implying a deteriorated resilience against magnetic field when the junction length is above this value. Moreover, only negligible fractions of switching current (less than 2%) systematically remain in the longer junctions—emphasizing their poor performance in magnetic fields. We emphasize that the particular shape of the dependence of the ratio on junction length could also vary depending on the choice of the normal conductance range and the subsequently determined gate intervals for averaging. However, the main qualitative features of such dependence would still remain. The impact of the junction length will be discussed in particular in the following paragraphs.

In Figures 3 and 4 two different approaches have been taken when quantifying the supercurrent resilience against magnetic field. Both approaches have led to the same observation—by reducing the junction length, supercurrent resilience against magnetic field can be significantly improved. This is a common and reproducible feature of the short JJs in our study. The observations still hold despite variations in the switching current dependences on the gate voltage or the parallel field. In the following two paragraphs, possible mechanisms for the length dependent supercurrent resilience are discussed.

The superconducting Al shell has a mean free path *l*_e_ of ∼0.9 nm according to a recent work,^26^ which uses the same machine for the Al growth. The extraordinarily short *l*_e_ in the thin Al shell is most likely due to massive surface scatterings and moderate nonuniformities. Together with a phase coherence length ξ_0_ of ∼1.6 μm from a bulk Al,^38^ the superconducting phase coherence length ξ of the Al shell in our work is estimated to be ∼38 nm with the formula  in the dirty superconductor limit.^39^ Then, JJs longer than ξ are in the long-junction limit and the superconducting proximity effect in these junctions is weakened in comparison with the short junctions. Then, weakened induced superconductivity in long junctions leads to a poor performance in magnetic fields. Destructive interference between transversal nanowire modes is considered as another dominant reason for reduced supercurrent critical field in longer junctions.^28^ The phase differences between modes can be accumulated in magnetic fields via either the Zeeman effect^1,2^ or the orbital effect.^40^ The Zeeman-induced phase accumulation is proportional to the Zeeman energy and the junction length,^1,2^ while the contribution from the orbital effect is proportional to the magnetic field and the junction length.^40^ Considering the large *g* factor in InSb (∼50^30,41^) and the relatively large magnetic field (∼0.5 T), significant phase accumulations are expected in long junctions. In this case, a prominent destructive interference is likely to appear in small magnetic fields for long junctions, resulting in reduced critical fields of supercurrent.

In this paragraph, we make a further analysis of other relevant effects, including a gate-tunable superconductor–semicondutor hybridization under superconducting shells, disorder, and spin–orbit interaction. The nine JJs are tuned by a global back gate, which at positive values may reduce the hybridization of the semiconductor under the superconducting leads.^37,42,43^ As shown in Figure S9, we have observed decreased induced superconducting gaps for long Josephson junctions, implying reduced semiconductor–superconductor couplings in these devices. This is likely due to a different gating effect on semiconductor–superconductor hybrids for different junctions. In order to investigate the relevance of such effect, we have measured an additional short JJ device (Device 10, the right arm of the SQUID device from Figure 5). This device utilizes a bottom gate under the junction and one bottom gate under each superconducting lead. Importantly, we find that applying a positive gate voltage locally under a single superconducting lead does not reduce the superconductor–semiconductor coupling to an extent that systematically limits the resilience of supercurrent (see Figure S10). The mean free path of the InSb nanowires is ∼300 nm,^41^ longer than all junctions. Thus, the influence of disorder is expected to be less important. A different spin–orbit interaction in different devices might happen, as gate voltages are not the same for all devices and different electric fields may be present in different junctions. The presence of spin–orbit interaction together with magnetic fields can lead to an anomalous superconducting phase,^1−3^ further complicating the interference effects, especially in long junctions.

**Figure 5:**
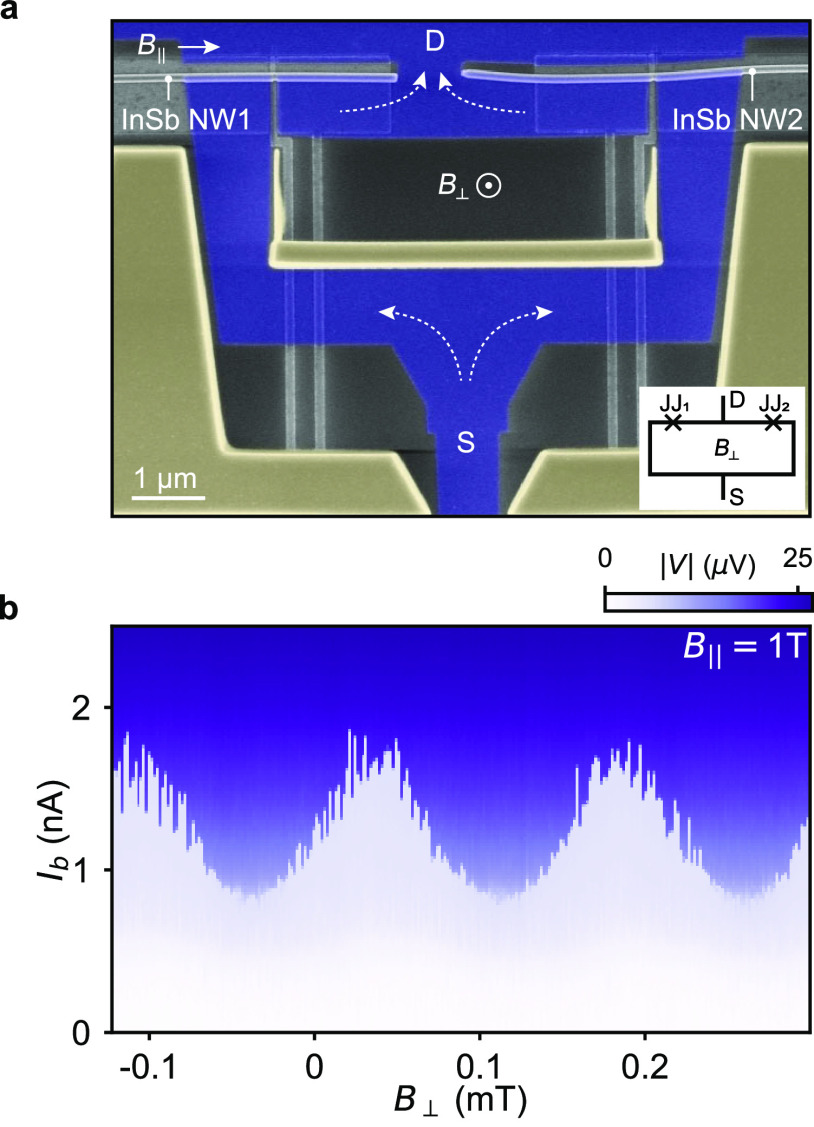
SQUID operating at a parallel magnetic field of 1 T. (a) False-colored SEM image of two hybrid 40 nm long InSb-Al nanowire Josephson junctions defined by the shadow walls (yellow). The two junctions enclose a superconducting Al (blue) loop in the SQUID architecture. A magnetic field *B*_∥_ is applied along two parallel InSb nanowires hosting the Josephson junctions. A perpendicular out-of-plane magnetic field *B*_⊥_ controls the magnetic flux through the superconducting loop between the source (S) and the drain (D). The inset image displays the equivalent device circuit. (b) Current bias measurement on the SQUID at the parallel magnetic field *B*_∥_ = 1 T showing oscillations of the SQUID switching current as the magnetic flux through the SQUID loop is swept by applying *B*_⊥_.

From the above results, we find that significantly reducing the nanowire JJ length is essential for preserving supercurrents in a high magnetic field. Here, we take a step further and incorporate the short nanowire JJs into a SQUID architecture. Figure 5a shows a false-colored SEM of a SQUID consisting of two 40 nm JJs formed in two parallel InSb nanowires. The shadow wall structure (yellow) is lithographically defined such that after the Al (blue) deposition two JJs enclose the superconducting loop denoted by the white arrows. Since the two arms are parallel, a magnetic field *B*_∥_ can be applied parallel to both JJs while the out-of-plane perpendicular magnetic field *B*_⊥_ is applied to sweep the flux threading the loop. Upon applying *B*_∥_ = 1 T, both junctions are independently tuned by the underlying local bottom gates to a finite supercurrent. As shown in Figure 5b, the oscillations of the switching current indicate a supercurrent interference persisting despite the high parallel field. In comparison with the previous work on nanowire SQUIDs,^3,44^ this observation of supercurrent interference at *B*_∥_ = 1 T represents a significant improvement of the SQUID field compatibility. The control and detection of the phase of supercurrent at high magnetic field is of crucial importance for studying various high-field-related phenomena in hybrid nanowire devices.^10,18,45,46^

In conclusion, we demonstrate that the length of a hybrid nanowire Josephson junction is an essential parameter that determines its supercurrent resilience against magnetic fields. Nanowire JJs with a length of less than 40 nm can be precisely defined by the shadow wall angle-deposition technique and are shown to reproducibly preserve supercurrent at parallel magnetic fields exceeding 1.3 T. A superconducting quantum interference device (SQUID) utilizing such junctions displays supercurrent interference at the parallel field of 1 T. Our study shows that hybrid nanowire Josephson junctions of significantly reduced junction length can be considered as necessary building blocks in various hybrid nanowire devices which exploit Josephson coupling at high magnetic field.

## Data Availability

Raw data and process files of this work are available at 10.5281/zenodo.7319481.

## Supporting Information Available

Details of device fabrications, measurement setups, and data analysis, the effect of junction length and global back gate on induced superconducting gap, and the influence of local gates on supercurrent resilience (PDF)

## References

[ref1] Yokoyama T.; Eto M.; Nazarov Y. V. Josephson Current through Semiconductor Nanowire with Spin–Orbit Interaction in Magnetic Field. J. Phys. Soc. Jpn. 2013, 82, 054703 10.7566/JPSJ.82.054703.

[ref2] Yokoyama T.; Eto M.; Nazarov Y. V. Anomalous Josephson effect induced by spin-orbit interaction and Zeeman effect in semiconductor nanowires. Phys. Rev. B 2014, 89, 195407 10.1103/PhysRevB.89.195407.

[ref3] Szombati D. B.; Nadj-Perge S.; Car D.; Plissard S. R.; Bakkers E. P. A. M.; Kouwenhoven L. P. Josephson ϕ_0_-junction in nanowire quantum dots. Nature Phys. 2016, 12, 568–572. 10.1038/nphys3742.

[ref4] Strambini E.; Iorio A.; Durante O.; Citro R.; Sanz-Fernández C.; Guarcello C.; Tokatly I. V.; Braggio A.; Rocci M.; Ligato N.; Zannier V.; Sorba L.; Bergeret F. S.; Giazotto F. A Josephson phase battery. Nat. Nanotechnol. 2020, 15, 656–660. 10.1038/s41565-020-0712-7. 32541945

[ref5] San-Jose P.; Prada E.; Aguado R. Mapping the topological phase diagram of multiband semiconductors with supercurrents. Phys. Rev. Lett. 2014, 112, 137001 10.1103/PhysRevLett.112.137001. 24745449

[ref6] Lutchyn R. M.; Sau J. D.; Sarma S. D. Majorana fermions and a topological phase transition in semiconductor-superconductor heterostructures. Phys. Rev. Lett. 2010, 105, 077001 10.1103/PhysRevLett.105.077001. 20868069

[ref7] Oreg Y.; Refael G.; von Oppen F. Helical liquids and Majorana bound states in quantum wires. Phys. Rev. Lett. 2010, 105, 177002 10.1103/PhysRevLett.105.177002. 21231073

[ref8] Schrade C.; Hoffman S.; Loss D. Detecting topological superconductivity with ϕ_0_-Josephson junctions. Phys. Rev. B 2017, 95, 195421 10.1103/PhysRevB.95.195421.

[ref9] Cayao J.; San-Jose P.; Black-Schaffer A. M.; Aguado R.; Prada E. Majorana splitting from critical currents in Josephson junctions. Phys. Rev. B 2017, 96, 205425 10.1103/PhysRevB.96.205425.

[ref10] Schrade C.; Fu L. Majorana Superconducting Qubit. Phys. Rev. Lett. 2018, 121, 267002 10.1103/PhysRevLett.121.267002. 30636155

[ref11] Cayao J.; Black-Schaffer A. M.; Prada E.; Aguado R. Andreev spectrum and supercurrents in nanowire-based SNS junctions containing Majorana bound states. Beilstein J. Nanotechnol. 2018, 9, 1339–1357. 10.3762/bjnano.9.127. 29977669 PMC6009489

[ref12] Chen C.-Z.; He J. J.; Ali M. N.; Lee G.-H.; Fong K. C.; Law K. T. Asymmetric Josephson effect in inversion symmetry breaking topological materials. Phys. Rev. B 2018, 98, 075430 10.1103/PhysRevB.98.075430.

[ref13] Turini B.; Salimian S.; Carrega M.; Iorio A.; Strambini E.; Giazotto F.; Zannier V.; Sorba L.; Heun S. Josephson Diode Effect in High-Mobility InSb Nanoflags. Nano Lett. 2022, 22, 8502–8508. 10.1021/acs.nanolett.2c02899. 36285780 PMC9650771

[ref14] Mazur G. P.; van Loo N.; van Driel D.; Wang J.-Y.; Badawy G.; Gazibegovic S.; Bakkers E. P. A. M.; Kouwenhoven L. P. The gate-tunable Josephson diode. arXiv preprint 2022, 2211.14283, 10.48550/arXiv.2211.14283 [Accessed: Nov 25, 2022].

[ref15] Yuan N. F. Q.; Fu L. Supercurrent diode effect and finite-momentum superconductors. Proc. Natl. Acad. Sci. U.S.A. 2022, 119, 2119548119 10.1073/pnas.2119548119. PMC9169709 35377813

[ref16] Davydova M.; Prembabu S.; Fu L. Universal Josephson diode effect. Sci. Adv. 2022, 8, eabo0309 10.1126/sciadv.abo0309. 35675396 PMC9176746

[ref17] Legg H. F.; Loss D.; Klinovaja J. Superconducting diode effect due to magnetochiral anisotropy in topological insulators and Rashba nanowires. Phys. Rev. B 2022, 106, 104501 10.1103/PhysRevB.106.104501.

[ref18] Souto R. S.; Leijnse M.; Schrade C. Josephson Diode Effect in Supercurrent Interferometers. Phys. Rev. Lett. 2022, 129, 267702 10.1103/PhysRevLett.129.267702. 36608204

[ref19] Wu H.; Wang Y.; Xu Y.; Sivakumar P. K.; Pasco C.; Filippozzi U.; Parkin S. S. P.; Zeng Y.-J.; McQueen T.; Ali M. N. The field-free Josephson diode in a van der Waals heterostructure. Nature 2022, 604, 653–656. 10.1038/s41586-022-04504-8. 35478238

[ref20] Pal B.; Chakraborty A.; Sivakumar P. K.; Davydova M.; Gopi A. K.; Pandeya A. K.; Krieger J. A.; Zhang Y.; Date M.; Ju S.; Yuan N.; Schroter N. B. M.; Fu L.; Parkin S. S. P. Josephson diode effect from Cooper pair momentum in a topological semimetal. Nat. Phys. 2022, 18, 1228–1233. 10.1038/s41567-022-01699-5. 36217362 PMC9537108

[ref21] Baumgartner C.; Fuchs L.; Costa A.; Reinhardt S.; Gronin S.; Gardner G. C.; Lindemann T.; Manfra M. J.; Junior P. E. F.; Kochan D.; Fabian J.; Paradiso N.; Strunk C. Supercurrent rectification and magnetochiral effects in symmetric Josephson junctions. Nat. Nanotechnol. 2022, 17, 39–44. 10.1038/s41565-021-01009-9. 34795437

[ref22] Zhang B.; Li Z.; Aguilar V.; Zhang P.; Pendharkar M.; Dempsey C.; Lee J.; Harrington S.; Tan S.; Meyer J.; Houzet M.; Palmstrøm C.; Frolov S. Evidence of 0-Josephson junction from skewed diffraction patterns in Sn-InSb nanowires. arXiv preprint 2022, 2212.00199, 10.48550/arXiv.2212.00199 [Accessed: Dec 1, 2022].

[ref23] Gül Ö.; et al. Hard superconducting gap in InSb nanowires. Nano Lett. 2017, 17, 2690–2696. 10.1021/acs.nanolett.7b00540. 28355877 PMC5446204

[ref24] Kanne T.; Marnauza M.; Olsteins D.; Carrad D. J.; Sestoft J. E.; de Bruijckere J.; Zeng L.; Johnson E.; Olsson E.; Grove-Rasmussen K.; Nygård J. Epitaxial Pb on InAs nanowires for quantum devices. Nat. Nanotechnol. 2021, 16, 776–781. 10.1038/s41565-021-00900-9. 33972757

[ref25] Pendharkar M.; et al. Parity-preserving and magnetic field–resilient superconductivity in InSb nanowires with Sn shells. Science 2021, 372, 508–511. 10.1126/science.aba5211. 33858990

[ref26] Mazur G. P.; et al. Spin-mixing enhanced proximity effect in aluminum-based superconductor–semiconductor hybrids. Adv. Mater. 2022, 34, 2202034 10.1002/adma.202202034. 35680622

[ref27] Heedt S.; et al. Shadow-wall lithography of ballistic superconductor–semiconductor quantum devices. Nat. Commun. 2021, 12, 4914 10.1038/s41467-021-25100-w. 34389705 PMC8363628

[ref28] Zuo K.; Mourik V.; Szombati D. B.; Nijholt B.; van Woerkom D. J.; Geresdi A.; Chen J.; Ostroukh V. P.; Akhmerov A. R.; Plissard S. R.; Car D.; Bakkers E. P. A. M.; Pikulin D. I.; Kouwenhoven L. P.; Frolov S. M. Supercurrent interference in few-mode nanowire Josephson junctions. Phys. Rev. Lett. 2017, 119, 187704 10.1103/PhysRevLett.119.187704. 29219554

[ref29] Doh Y.-J.; Dam J. A. V.; Roest A. L.; Bakkers E. P. A. M.; Kouwenhoven L. P.; Franceschi S. D. Tunable Supercurrent Through Semiconductor Nanowires. Science 2005, 309, 272–275. 10.1126/science.1113523. 16002611

[ref30] Nilsson H. A.; Samuelsson P.; Caroff P.; Xu H. Q. Supercurrent and Multiple Andreev Reflections in an InSb Nanowire Josephson Junction. Nano Lett. 2012, 12, 228–233. 10.1021/nl203380w. 22142358

[ref31] Abay S.; Persson D.; Nilsson H.; Xu H. Q.; Fogelström M.; Shumeiko V.; Delsing P. Quantized Conductance and Its Correlation to the Supercurrent in a Nanowire Connected to Superconductors. Nano Lett. 2013, 13, 3614–3617. 10.1021/nl4014265. 23898893

[ref32] Krogstrup P.; Ziino N. L. B.; Chang W.; Albrecht S. M.; Madsen M. H.; Johnson E.; Nygård J.; Marcus C. M.; Jespersen T. S. Epitaxy of semiconductor–superconductor nanowires. Nat. Mater. 2015, 14, 400–406. 10.1038/nmat4176. 25581626

[ref33] Carrad D. J.; Bjergfelt M.; Kanne T.; Aagesen M.; Krizek F.; Fiordaliso E. M.; Johnson E.; Nygård J.; Jespersen T. S. Shadow Epitaxy for In Situ Growth of Generic Semiconductor/Superconductor Hybrids. Adv. Mater. 2020, 32, 1908411 10.1002/adma.201908411. 32337791

[ref34] Borsoi F.; et al. Single-shot fabrication of semiconducting–superconducting nanowire devices. Adv. Funct. Mater. 2021, 31, 2102388 10.1002/adfm.202102388.

[ref35] Badawy G.; Gazibegovic S.; Borsoi F.; Heedt S.; Wang C.-A.; Koelling S.; Verheijen M. A.; Kouwenhoven L. P.; Bakkers E. P. A. M. High mobility stemless InSb nanowires. Nano Lett. 2019, 19, 3575–3582. 10.1021/acs.nanolett.9b00545. 31094527

[ref36] de Moor M. W. A.; et al. Electric field tunable superconductor-semiconductor coupling in Majorana nanowires. New J. Phys. 2018, 20, 103049 10.1088/1367-2630/aae61d.

[ref37] Shen J.; et al. Full parity phase diagram of a proximitized nanowire island. Phys. Rev. B 2021, 104, 045422 10.1103/PhysRevB.104.045422.

[ref38] Kittel C. Introduction to Solid State Physics, 8th ed.; Wiley: 2004.

[ref39] Tinkham M. Introduction to Superconductivity, 2nd ed.; McGraw-Hill: 1996.

[ref40] Gharavi K.; Baugh J. Orbital Josephson interference in a nanowire proximity-effect junction. Phys. Rev. B 2015, 91, 245436 10.1103/PhysRevB.91.245436.

[ref41] van Weperen I.; Plissard S. R.; Bakkers E. P. A. M.; Frolov S. M.; Kouwenhoven L. P. Quantized Conductance in an InSb Nanowire. Nano Lett. 2013, 13, 387–391. 10.1021/nl3035256. 23259576

[ref42] Mikkelsen A. E.; Kotetes P.; Krogstrup P.; Flensberg K. Hybridization at superconductor-semiconductor interfaces. Phys. Rev. X 2018, 8, 031040 10.1103/PhysRevX.8.031040.

[ref43] Antipov A. E.; Bargerbos A.; Winkler G. W.; Bauer B.; Rossi E.; Lutchyn R. M. Effects of gate-induced electric fields on semiconductor Majorana nanowires. Phys. Rev. X 2018, 8, 031041 10.1103/PhysRevX.8.031041.

[ref44] Wang J.-Y.; et al. Supercurrent parity meter in a nanowire Cooper pair transistor. Sci. Adv. 2022, 8, eabm9896 10.1126/sciadv.abm9896. 35452283 PMC9032955

[ref45] Liu C.-X.; van Heck B.; Wimmer M. Josephson current via an isolated Majorana zero mode. Phys. Rev. B 2021, 103, 014510 10.1103/PhysRevB.103.014510.

[ref46] Schrade C.; Fu L. Parity-controlled 2π Josephson effect mediated by Majorana Kramers pairs. Phys. Rev. Lett. 2018, 120, 267002 10.1103/PhysRevLett.120.267002. 30004723

