# Supplementary material for: Impact of Junction Length on Supercurrent Resilience against Magnetic Field in InSb-Al Nanowire Josephson Junctions
Source: Nano Lett. 2023 May 22;23(11):4716–22. doi: 10.1021/acs.nanolett.2c04485 (PMC10273483; doi:10.1021/acs.nanolett.2c04485)
Supplement: Supplementary file 1 — nl2c04485_si_001.pdf [file nl2c04485_si_001.pdf]

Supporting Information: Impact of junction length on supercurrent resilience against magnetic field in InSb-Al nanowire Josephson junctions

Vukan Levajac,[*,†] Grzegorz P. Mazur,[†] Nick van Loo,[†] Francesco Borsoi,[†] Ghada Badawy,[‡] Sasa Gazibegovic,[‡] Erik P. A. M. Bakkers,[‡] Sebastian Heedt,[†] Leo P. Kouwenhoven,[†] and Ji-Yin Wang[*,†,¶]

†*QuTech and Kavli Institute of Nanoscience, Delft University of Technology, 2600 GA Delft, The Netherlands*

‡*Department of Applied Physics, Eindhoven University of Technology, 5600 MB Eindhoven, The Netherlands*

¶*Beijing Academy of Quantum Information Sciences, 100193 Beijing, China (current address)*

E-mail: v.levajac@tudelft.nl; wangjiyinshu@gmail.com

Methods

The study in the main text is based on nine InSb-Al nanowire Josephson junction (JJ) devices (Device 1-9) and one InSb-Al nanowire superconducting quantum interference device (SQUID). The JJ devices are used to investigate the impact of junction length on the supercurrent resilience against magnetic field. The SQUID is used to demonstrate how JJs

hosting resilient supercurrent can be embedded into a superconducting loop to yield supercurrent interference at high magnetic field. As an additional measurement, the supercurrent resilience against magnetic field is examined in an additional JJ device (Device 10), that is the single arm of the SQUID.

Device fabrication

All devices in this work were fabricated on p^+-doped Si wafers covered with $\sim 300\,$nm of thermal SiO_2. For Device 1-9, the thermal SiO_2 is used as a global back gate dielectric. For the SQUID, extra steps in the substrate fabrication were taken in order to create local bottom gates. On top of the thermal SiO_2, the local bottom gates were lithographically defined and produced by depositing 3/17 nm of Ti/Pd by electron beam evaporation. Then, $\sim 20\,$nm of high-quality HfO_2 layer was grown by atomic layer deposition (ALD) at 110 °C to act as the bottom gate dielectric.

Dielectric structures corresponding to specific shadow-wall patterns were defined by electron-beam lithography on top of the thermal SiO_2 and ALD HfO_2 for the Device 1-9 and the SQUID, respectively. Namely, FOx-25 (HSQ) was spun at 1.5 krpm for one minute, followed by 2 minutes of hot baking at 180 °C and patterning lithographically. The HSQ is then developed with MF-321 at 60 °C for 5 minutes and the substrates are subsequently dried using critical point dryer. This step was followed by the nanowire deposition by an optical nanomanipulator setup and the stemless InSb nanowires[1] were precisely placed on top of the global back gate (Device 1-9) or the array of local bottom gates (SQUID), close to the HSQ structures.

Deposition of the superconducting Al film was carried out in the nominally identical steps for all devices in this study. After gentle hydrogen cleaning of the nanowire surface, the superconducting film was grown by directional evaporation of Al. The Al flux in the deposition was 17 nm and the angle with respect to the substrate was 30°[2,3]. Due to the specific angle and the regular hexagonal nanowire cross-section, the Al film continuously

covers three nanowire facets, as shown in the above cited references. On one facet the Al film is deposited perpendicularly and the film thickness on this facet is $\sim 15\,\text{nm}$, as $\sim 2\,\text{nm}$ of Al self-terminately oxidizes in the air. The direction of the Al deposition forms an angle of 30° with the other two facets and these two facets therefore receive $\sin 30° = 0.5$ of the Al flux and have the film thickness of $\sim 7\,\text{nm}$ after the oxidation. Lithographically patterned dielectric structures cast shadows during the Al deposition and therefore selectively define the sections along the nanowire where the superconducting film is grown and where the semiconducting junction is formed. Additionally, the arrangement of the shadow-wall structures on the SQUID substrate determines a shadowed substrate area without Al enclosed by the two JJs that represents the superconducting loop of the SQUID. Finally, in all devices the superconducting film on the nanowire facets forms a continuous connection to the substrate and extends to pre-patterned bonding pads such that additional fabrication steps to contact the nanowires are not needed.

In this work, seven nanowire JJ devices (Device 1-7) were fabricated on a single chip, while the other two (Device 8-9) come from other two chips that passed through the nominally identical fabrication steps. The SQUID was fabricated on a separate chip in the fabrication steps as explained above.

Measurement setup

We perform the electrical transport measurements at $\sim 20\,\text{mK}$ base temperature in a dilution refrigerator equipped with a vector-rotate magnet. Source and drain leads of the device are bonded each to two printed circuit board (PCB) pads that are via low-pass filters connected to the fridge lines. In this way each device occupies in total four fridge lines - allowing for measurements in a two- and four-terminal configuration.

We perform the conductance measurements in the two-terminal voltage bias setup in the standard lock-in configuration. Source and drain are connected to the measurement setup by two fridge lines, while the remaining two fridge lines are kept floated. The voltage bias V_b

is swept by a dc-voltage source while the ac-voltage $dV_b = 10\mu V$ is set by a lock-in amplifier. The total current $I + dI$ through the sample is measured by a current-meter amplifier. The dc- and the ac-voltage drops over the sample are obtained by subtracting the voltage drops over the series resistance $R_s = 8.89k\Omega$ as $V = V_b - IR_s$ and $dV = dV_b - dIR_s$. This series resistance accounts for other resistive elements in the circuit such as the two fridge lines, the resistance of the voltage source and the current-meter amplifier and the resistance of the low-pass filters on the printed circuit board. For collecting the data from which the switching current is extracted, four-terminal current-bias setup is used. Two fridge lines are used to connect a current source and apply the dc-current bias I_b through a device, while the other two fridge lines are used to connect a voltage-meter and measure the dc-voltage drop V over the device. The current bias is swept in steps of 20 pA - 60 pA, depending on the range of current-bias that is applied. As the voltage-meter measures at the room temperature the sum of the voltage drops over the device and the two fridge lines, a dc-offset of $\sim 0.01\,\mathrm{mV}$ are substracted to compensate for the difference in the thermal voltage drops over the fridge lines.

Data analysis

All the codes used for the data analysis in this work are available in the data repository. The details of the data analysis procedures performed in these codes are described in the following subsections.

Extracting normal state conductance G_n

Normal state conductance G_n is extracted from the data collected in the voltage-bias measurements of the nanowire JJ devices. After correcting for the series resistance R_s (as explained in the previous section), the normal state conductance is obtained as $G_n(V_g) = (G_n^+(V_g)+G_n^-(V_g))/2$ where $G_n^+(V_g) = \langle \frac{dI}{dV}(V_g, 1\,\mathrm{mV} < V < 2\,\mathrm{mV})\rangle$ and $G_n^-(V_g) = \langle \frac{dI}{dV}(V_g, -2\,\mathrm{mV} < V < -1\,\mathrm{mV})\rangle$ are averaged conductances at the positive and the negative source-drain volt-

ages much larger than the double value of the superconducting gap ($2\Delta \sim 500\,\mu$V).

Extracting switching current I_{sw}

Switching current is extracted for each (V, I_b) trace measured in the current-bias setup. Four-examples of (V, I_b) traces are shown in the top parts of Fig. S1a-d. The corresponding differential resistance $(dV/dI_b, I_b)$ traces are calculated as numerical derivatives and are plotted in the bottom parts of Fig. S1a-d. The data in Fig. S1 corresponds to four traces from the back gate sweep at parallel magnetic field of 1 T in Device 2. These traces are chosen to motivate the particular method used in the switching current extraction.

From a perfectly clean (V, I_b) trace, as the one in Fig. S1a, with a single voltage step corresponding to the switching current I_{sw}, I_{sw} can in principle be extracted by setting a threshold voltage V_{th}, such that $V_{th} = V(I_b = I_{sw})$. However, this can give underestimated extracted values as the voltage V can due to noise fluctuate for current bias values lower than the switching current - as shown in the (V, I_b) trace in Fig. S1b. Setting higher V_{th} values to prevent this, can, on the other hand, give an overestimation of the extracted value if the switching current is small. Therefore, when extracting I_{sw}, we rather look at the maximum in the differential resistance, as it resembles the sharpness of a switch in a (V, I_b) trace.

For each differential resistance $(dV/dI_b, I_b)$ trace, the maximal value (peak) of dV/dI_b is found and divided by the third value of the same $(dV/dI_b, I_b)$ trace sorted in decreasing order. In this way we quantify how dominant the peak in the differential resistance is. If the obtained value is smaller than the analogous value obtained from the trace in Fig. S1d with clearly no switch in it - the peak in differential resistance is not dominant and the switching current is extracted as a "not a number" (NaN) value. These NaN values correspond to the interruptions in the red I_{sw} traces plotted over 2D maps throughout the study.

The trace in Fig. S1c depicts that the range over which the dominant peak in $(dV/dI_b, I_b)$ is searched for can affect the extracted value. For example, there is a dominant peak in $(dV/dI_b, I_b)$ in Fig. S1c at $I_b \sim 1.7\,$nA, but it does not correspond to the switching current.

Therefore, the range in which the switching current is searched for is an important input parameter that is marked by the blue lines in Fig. S1. This parameter is commonly set at sufficiently high values and subsequently adjusted for particular traces where it leads to mistakes as the one described in Fig. S1c. The red lines in Fig. S1 mark the extracted switching current values and nicely match the dominant peaks of the differential resistance in the relevant ranges of the current bias.

The described algorithm successfully identifies the switching current in most of the traces. After applying it, additional corrections were made after checking how an extracted I_{sw} value matches to its corresponding (V, I_b) trace. Some extracted finite I_{sw} values were set then to NaN if found to have been extracted in a highly smeared (V, I_b) trace. On the other hand, in some non-smeared (V, I_b) traces with NaN extracted I_{sw} values, the switching current is re-extracted by extracting the position of the global maximum in the differential resistance trace. Such post-extraction corrections were performed equally frequently for all devices (5-10% of all (V, I_b) traces).

Extracting critical magnetic field B_{Ic}

By applying the above described algorithm to extract the switching current I_{sw}, we extract $I_{sw}(B)$ from the 2D maps shown in Fig. S7 where the voltage drop V is measured as the current bias I_b and the parallel magnetic field B are swept. By analyzing the evolution of the (V, I_b) linecuts in B field, it can be noticed that the algorithm may give an isolated NaN value for I_{sw} at some B value even if the switching current is correctly extracted at higher fields. Therefore, defining the critical field of switching current B_{Ic} as the lowest B field for which the algorithm gives NaN value for I_{sw} can lead to underestimations of B_{Ic}. However, if the algorithm gives NaN values for two consecutive B field values, then even occasionally extracted I_{sw} values different from NaN at higher fields are most often false-positive extracted values. We therefore determine the critical field B_{Ic} as the lowest field such that two consecutive extracted values for I_{sw} are NaN. In Fig. S7 I_{sw} is plotted up to

the determined B_{Ic} while the entire $I_{sw}(B)$ data is available in the data repository.

Effects of junction length and global back gate on induced superconducting gap

In order to measure the induced superconducting gap for Device 1-9 and study its evolution in parallel magnetic field, tunneling spectroscopy is performed in the voltage bias setup.

In Fig. S9 the evolution of induced superconducting gap in parallel magnetic field is shown for Device 1-9. Each subfigure represents a 2D map of the tunneling conductance as a function of the voltage drop over the junction and the parallel field. Two coherence peaks corresponding to the double value of the induced gap Δ appear in the tunneling conductance at $|V| = 2\Delta$. By extracting the peak separation and dividing it by 4 for each Device 1-9 at zero field, the values for induced superconducting gap are calculated. These values are shown as insets in Fig. S9, together with the global back gate voltage at which the corresponding conductance maps are obtained.

In Fig. S9 it can be seen that the three short junctions (Device 1,2 and 3) have larger values of the induced gap with the critical parallel field of $\sim 1.5\,\mathrm{T}$ - similar to the parent superconducting gap in the Al film[2–4]. On the other hand, the two longest junctions (Device 6 and 7) are characterized by reduced induced gaps and subgap states evolving towards zero energy and effectively closing the gap well before the parent superconducting gap vanishes. These differences in the induced gap sizes and their evolution in parallel magnetic field for junctions of different lengths are accompanied by differences in the gate settings at which different devices are set into the tunneling regime. Namely, it can be noticed that shorter devices mostly require low or even negative back gate voltages for reaching the tunneling regime, while this value is higher for the longer junctions. A valid question that arises is whether the differences in the tunneling spectroscopy in Fig. S9 are due to the differences in the junction lengths or due to the differences in the electrical fields induced by the different

gate voltages.

Despite the differences present among the nine devices in the tunneling regime regarding the back gate settings, the junction lengths and the conductance values, some conclusions can be made by looking at specific subsets of the devices for which some of these parameters are comparable. By comparing the data for Device 4, 5 and 7, it can be seen that with almost the same gate settings of $V_g \sim 2.15\,\mathrm{V}$ and the comparable tunneling conductance values $G_n \sim 0.3 - 0.4\,G_0$, the shortest device out of the three (Device 4) exhibits the largest induced gap that closes at the highest field. The data for the other two devices (Device 5 and 7) suggest that gradual increases of the junction length lead to weaker proximity effect with gradually smaller induced gap and gradually lower critical parallel field of the induced gap. Furthermore, the shortest device in the study (Device 8) requires the largest gate voltage to be tuned into the tunneling regime ($V_g = 5.7\,\mathrm{V}$) and still exhibits larger induced gap than the longest devices (Device 6-7) measured at the lower gate voltages. Despite the high gate voltage, the induced gap of Device 8 closes at $\sim 1.3\,\mathrm{T}$. However, in comparison to the remaining short junctions measured at significantly lower gate voltages (Device 1,2 and 3), Device 8 has poorer induced superconducting properties, probably due to the the high gate voltage and reduced superconductor-semiconductor coupling.

We can conclude that junction length is an important parameter that influences the induced superconducting gap. This does not exclude an effect that the applied back gate voltage has on induced superconductivity. Moreover, the data in Fig. S9 demonstrates that both the junction length and the back gate voltage determine the semiconductor-superconductor hybridization. This confirms that the electrostatic profile inside a hybrid nanowire JJ device - influenced by both device geometry and gate voltage - can control the strength of the semiconductor-superconductor hybridization[5,6].

The band offset at an InSb-Al interface can cause a bending of the InSb conduction band and results in a proximitized electron layer at the interdace with Al. Because of a finite lateral extension of such layers from the two sides of a short JJ, the junction superconducting

properties could be enhanced. Note that in some short JJs in our study the normal conductance and supercurrent have been measured to be finite when no back gate voltage is applied (see the data for Device 2 and 3 in Fig. S4). This could suggest that the accumulation layers can fully extend over a $\sim 30\,\mathrm{nm}$ junction by extending $\sim 15\,\mathrm{nm}$ laterally at each side.

The evidence of different strengths of hybridization in junctions of different lengths is in agreement with the reported zero-field values of the induced gap in Fig. S9 and the average switching current values at zero field in Fig. S4. Although the induced gap is characterized in the tunneling regime with no supercurrent, the critical parallel fields of switching current in Fig. 3e in the main text roughly match the parallel field values at which the induced gaps close in Fig. S9.

Effects of local gates on supercurrent resilience

As an additional measurement, we perform current bias measurements on a single Josephson junction (Device 10) which is one arm of the SQUID (see Fig. S10a and the Fabrication section for the details on the device design). The local bottom gates under the nanowire in Device 10 allow for a local tuning of the electro-chemical potential in different sections of the nanowire and can therefore serve to evaluate the effects of the local gating on the supercurrent resilience.

We perform current bias measurements on Device 10 while the other arm of the SQUID is pinched-off. The three bottom gates - TG and SG1/SG2 - approximately align with the junction and the superconducting leads, as shown in Fig. S10a. Two bottom gate voltages V_{SG1} and V_{SG2} mainly tune the nanowire sections covered by the superconductor, while the middle gate voltage V_{TG} mainly tunes the semiconducting junction. In this way the electro-chemical potential in the nanowire can be locally controlled, which is not possible in the global back gate configuration of nanowire JJ devices (Device 1-9) in the main text.

The dielectric used for the local bottom gates is ALD HfO_2 of $\sim 20\,\mathrm{nm}$ thickness. As

a comparison, the global back gate of the Device 1-9 utilizes thermal SiO_2 of $\sim 300\,\text{nm}$ thickness. By taking into account the dielectric constant values of HfO_2 and SiO_2 to be ~ 10 and ~ 4, respectively, the gating effect of the local bottom-gates is estimated to be at least 30 times larger than that of the global back gate.

In Fig. S10b-e, dependences of the extracted switching current I_{sw} (red) on a single bottom gate voltage are shown, while the other two bottom gates and the parallel magnetic field are fixed. By comparing Fig. S10b and Fig. S10c, it can be noticed that sweeping just V_{TG} qualitatively resembles the case when the global back-gate is swept (Fig. 1b, Fig. S4 and Fig. S8). When V_{SG1} and V_{SG2} are decreased in Fig. S10c in comparison to Fig. S10b, a slight decrease in I_{sw} can be observed. This can be attributed to V_{SG1} and V_{SG2} cross-coupling to the junction and effectively reducing its transmission. By looking at Fig. S10d and Fig. S10e, it can be seen that sweeping a single local bottom gate under the superconducting leads over $4.5\,\text{V}$ does not systematically affect the extracted switching current I_{sw}. In some cases, a slight increase in the background value of I_{sw} can be observed as V_{SG1} or V_{SG2} increase over $4.5\,\text{V}$ voltage range. This is also in agreement with V_{SG1} and V_{SG2} cross-coupling to the junction.

The fluctuations of the switching current magnitude in the single local bottom gate sweeps in Fig. S10b-e are comparable to those observed in the global back gate traces in the main text. Therefore, it cannot be determined whether the fluctuations in the back gate sweeps arise from the modulations of the electro-chemical potential of the junction or the nanowire sections under the superconducting leads. Importantly, we observe that applying positive voltage on the single local bottom gate under the superconducting lead does not diminish the semiconductor-superconductor coupling to an extent that the supercurrent of Device 10 is systematically suppressed.

Data selection and reproducibility

By systematically sweeping the back gate voltage V_g when measuring Device 1-7, we could identify the resilient gate settings $V_{g,res}$, as described in the main text. However, at the initial phase of the study, when measuring the chips from which Device 8-9 originate, the resilience of supercurrent against magnetic field was only examined at $V_g = 15\,\mathrm{V}$. Therefore, for these devices the identification of the resilient gate setting $V_{g,res}$ (like those shown in Fig. S6) was not performed. Still, we include Device 8-9 in our study as they manifest resilient supercurrent even at $V_g = 15\,\mathrm{V}$ which is not necessarily their $V_{g,res}$. Other short junction devices from these chips did not manifest such resilient supercurrent (critical parallel field of $\sim 0.7\,\mathrm{T}$ at $V_g = 15\,\mathrm{V}$) and long junction devices from these chips showed very poor supercurrent resilience (critical parallel field of $\sim 0.4\,\mathrm{T}$ at $V_g = 15\,\mathrm{V}$). We do not include these devices in our study as their critical parallel fields at $V_g = 15\,\mathrm{V}$ may be significantly smaller in comparison to their critical fields at the back gate tuned to their $V_{g,res}$ settings.

Importantly, we have never measured any long junction device (with or without back gate tuning) that showed better supercurrent resilience than the long junction devices (Device 6-7) presented in the study.

References

(1) Badawy, G.; Gazibegovic, S.; Borsoi, F.; Heedt, S.; Wang, C.-A.; Koelling, S.; Verheijen, M. A.; Kouwenhoven, L. P.; Bakkers, E. P. A. M. High mobility stemless InSb nanowires. *Nano Lett.* **2019**, *19*, 3575–3582.

(2) Heedt, S. et al. Shadow-wall lithography of ballistic superconductor–semiconductor quantum devices. *Nat. Commun.* **2021**, *12*, 4914.

(3) Borsoi, F. et al. Single-shot fabrication of semiconducting–superconducting nanowire devices. *Adv. Func. Mater.* **2021**, p. 2102388.

(4) Mazur, G. P. et al. Spin-mixing enhanced proximity effect in aluminum-based superconductor–semiconductor hybrids. *Adv. Mater.* **2022**, *34*, 1–8.

(5) de Moor, M. W. A. et al. Electric field tunable superconductor-semiconductor coupling in Majorana nanowires. *New J. Phys.* **2018**, *20*, 103049.

(6) Shen, J. et al. Full parity phase diagram of a proximitized nanowire island. *Phys. Rev. B* **2021**, *104*, 045422.

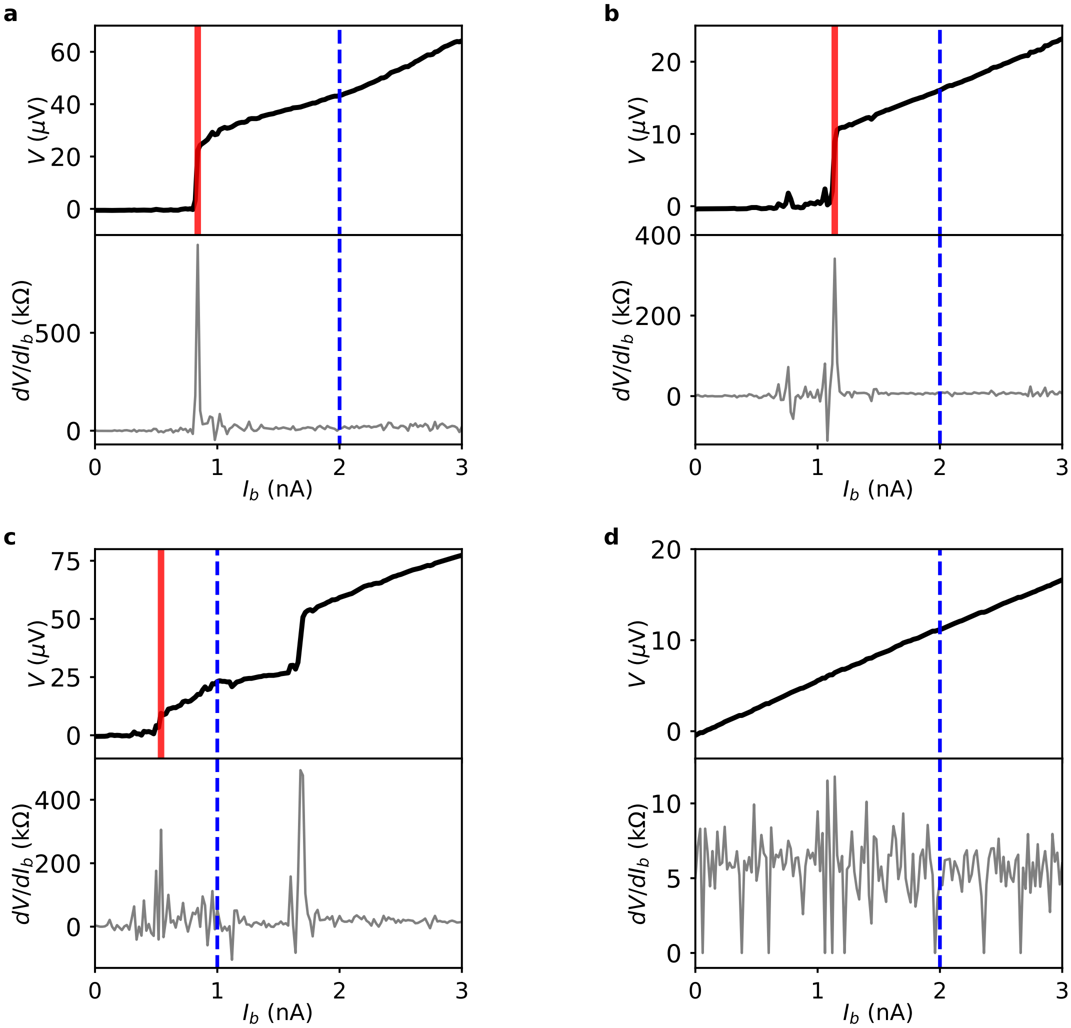

Figure S1: **Extraction of switching current:** Examples in **(a)-(d)** show the voltage drop (top) and the numerically calculated differential resistance (bottom) traces as functions of the current bias I_b. The extracted switching current I_{sw} (red) and the ranges over which the presence of a switch is examined (blue) are marked by the vertical lines. These traces were taken in Device 2 ($L = 31\,\mathrm{nm}$) at $B = 1\,\mathrm{T}$ parallel magnetic field.

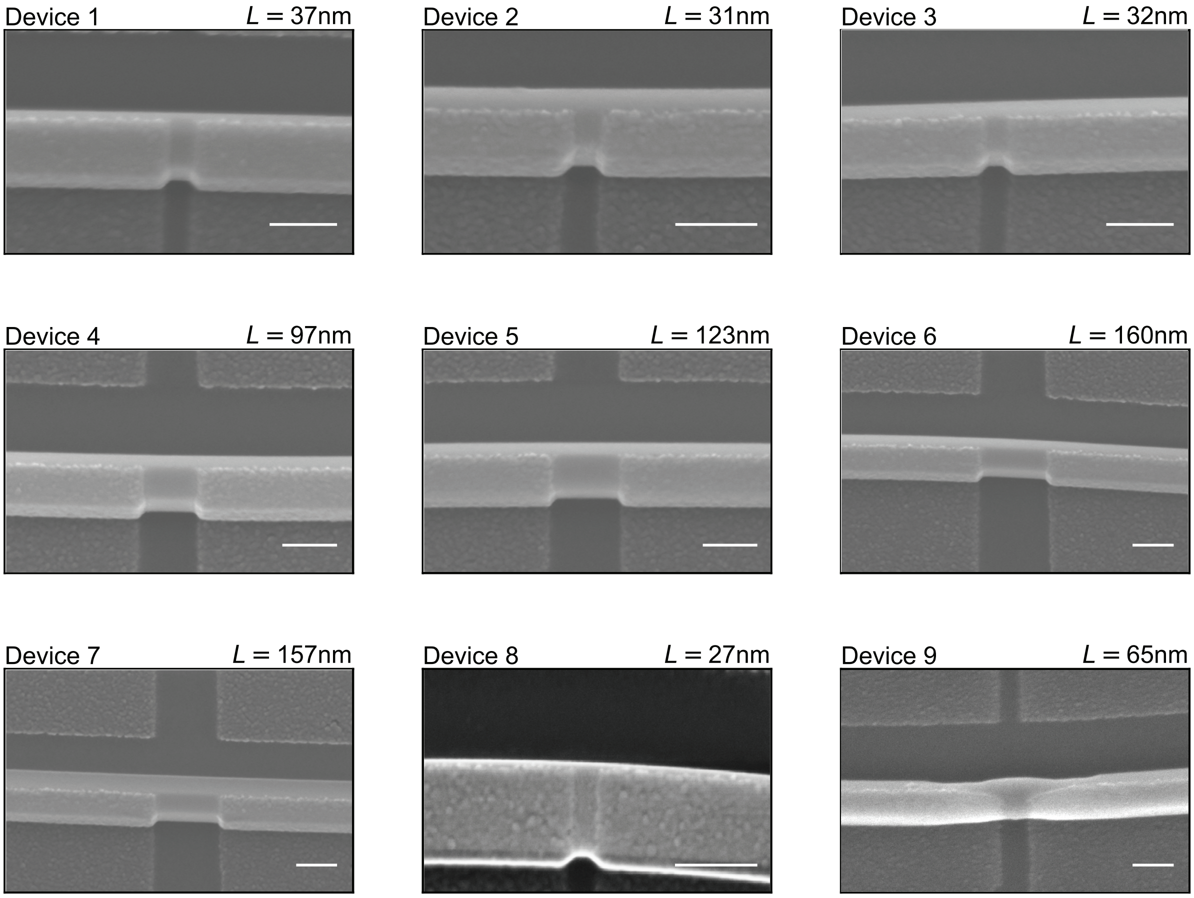

Figure S2: **Nine nanowire Josephson junction devices:** SEM images of the junctions with the corresponding device name (Device 1-9) and the junction length L. The diameter of the nanowires is $\sim 100\,\mathrm{nm}$ (between $90\,\mathrm{nm}$ and $110\,\mathrm{nm}$). The scale bars correspond to $100\,\mathrm{nm}$.

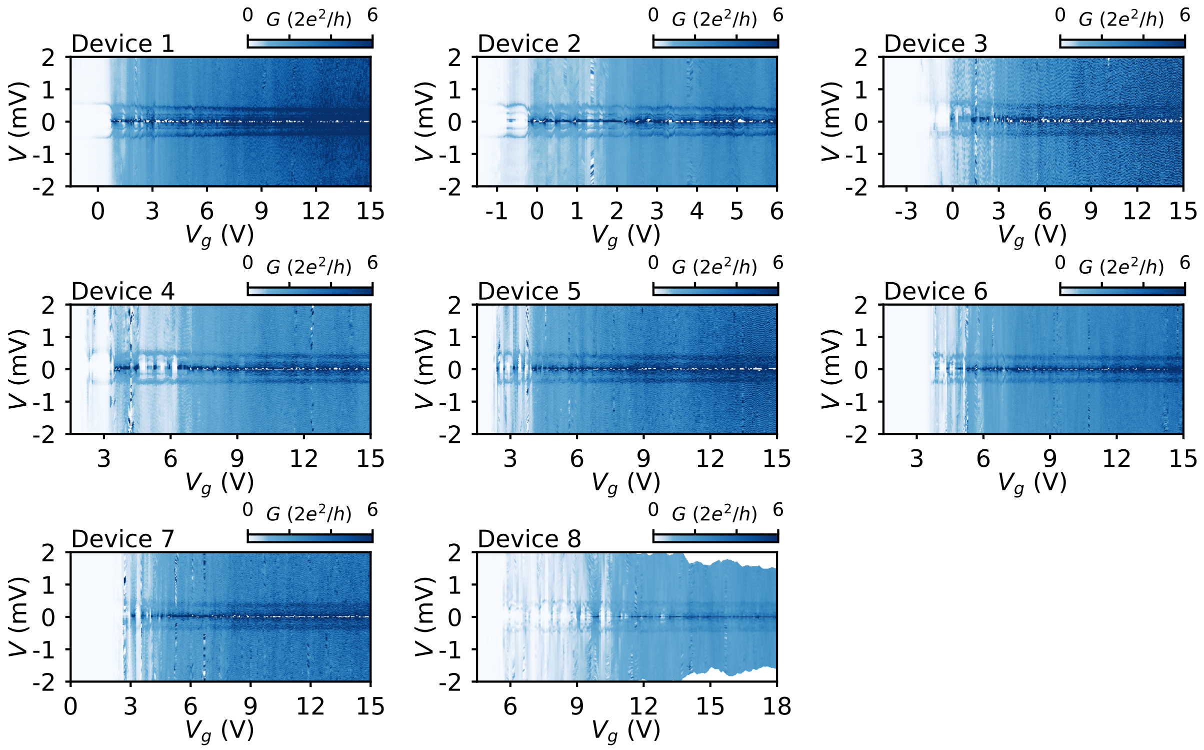

Figure S3: **Differential conductance at zero-field:** Measured differential conductance G through Device 1-8 as a function of the voltage drop V between the source and drain and the back gate voltage V_g. The normal state conductance dependences $G_n(V_g)$ for Device 1-8 are obtained from these 2D maps. as described in the Data analysis section. The analogous 2D map was not taken for Device 9 and the $G_n(V_g)$ dependence for this device was measured as a single trace at $V > 1\,\text{mV}$.

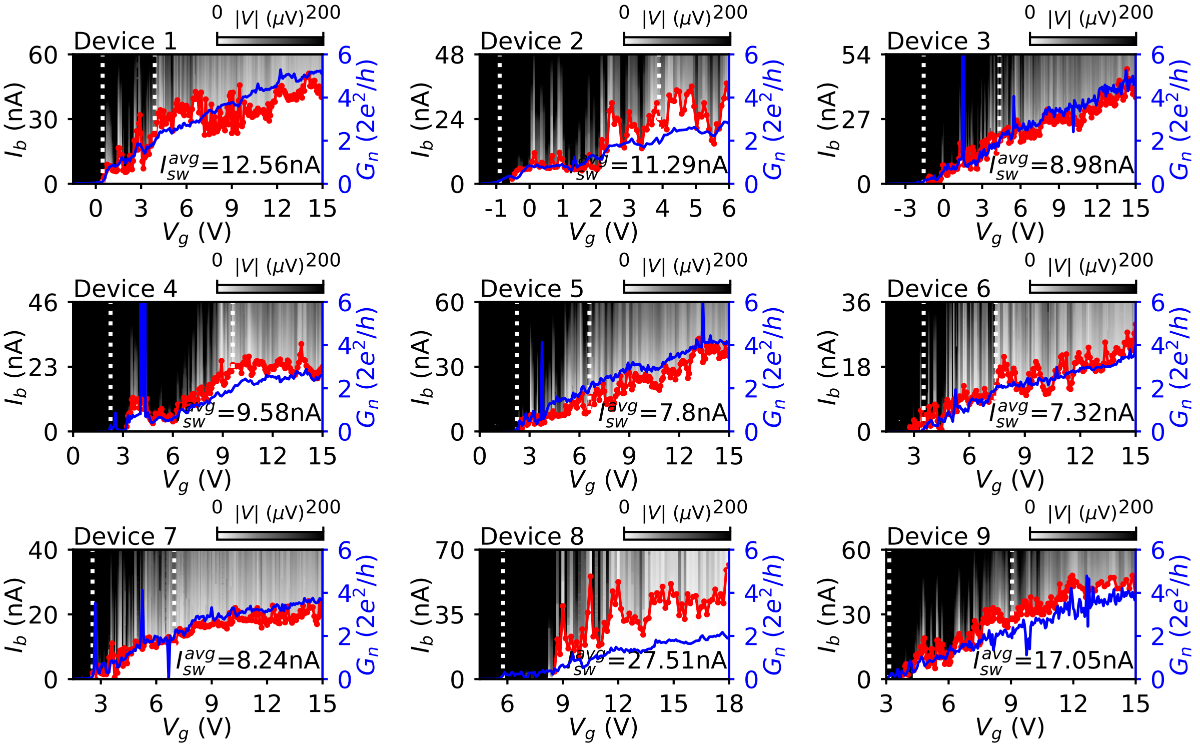

Figure S4: **Tunable switching current and normal conductance at zero-field:** - For Device 1-9 the extracted switching current I_{sw} (red) and measured normal conductance G_n (blue) are plotted over $I_b - V_g$ 2D maps obtained in the current bias measurements at zero-field. All devices show tunability by the back gate voltage V_g from the pinch-off regime with no supercurrent to the open regime with I_{sw} of several tenths of nA and G_n of few G_0 (with $G_0 = 2e^2/h$). $G_n(V_g)$ dependences are obtained from the data shown in Fig. S3. The white dotted vertical lines mark the ranges of V_g over which G_n increases from $0.1G_0$ to $2G_0$. The average switching currents in these intervals are shown as insets.

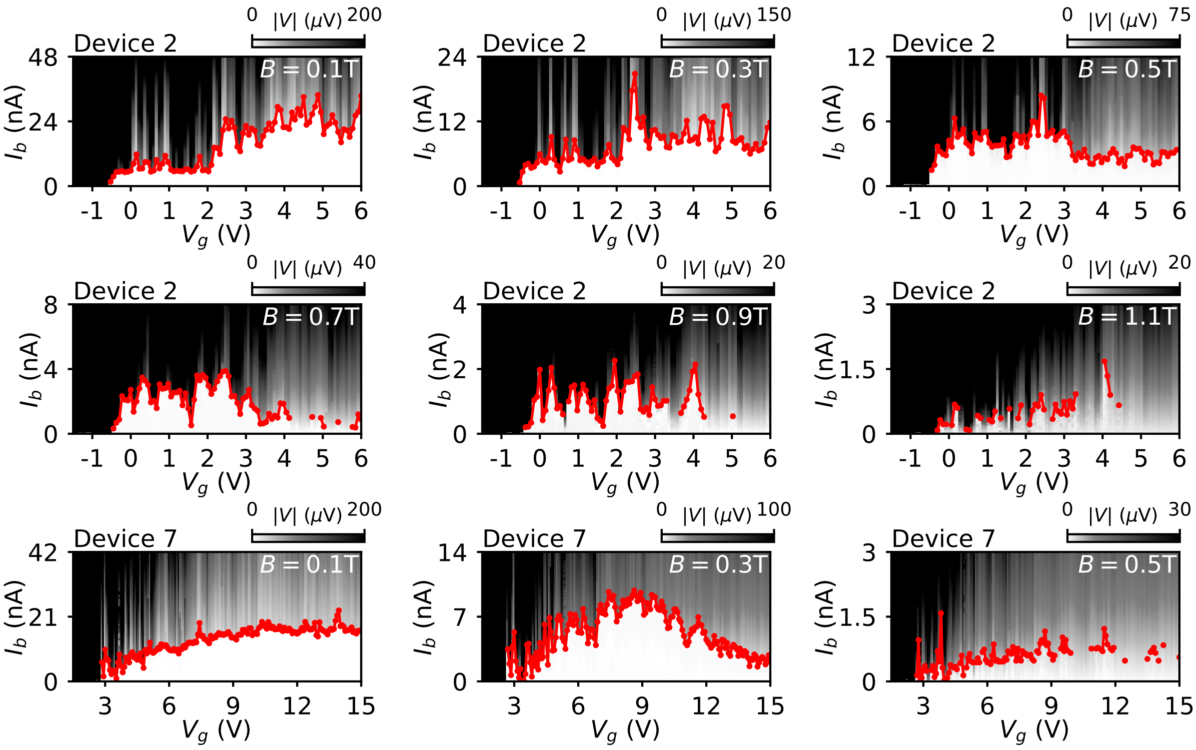

Figure S5: **Background data for Fig. 2:** The extracted switching current I_{sw} (red) as a function of the back gate voltage V_g at several parallel field values. The corresponding parallel magnetic fields are shown as insets.

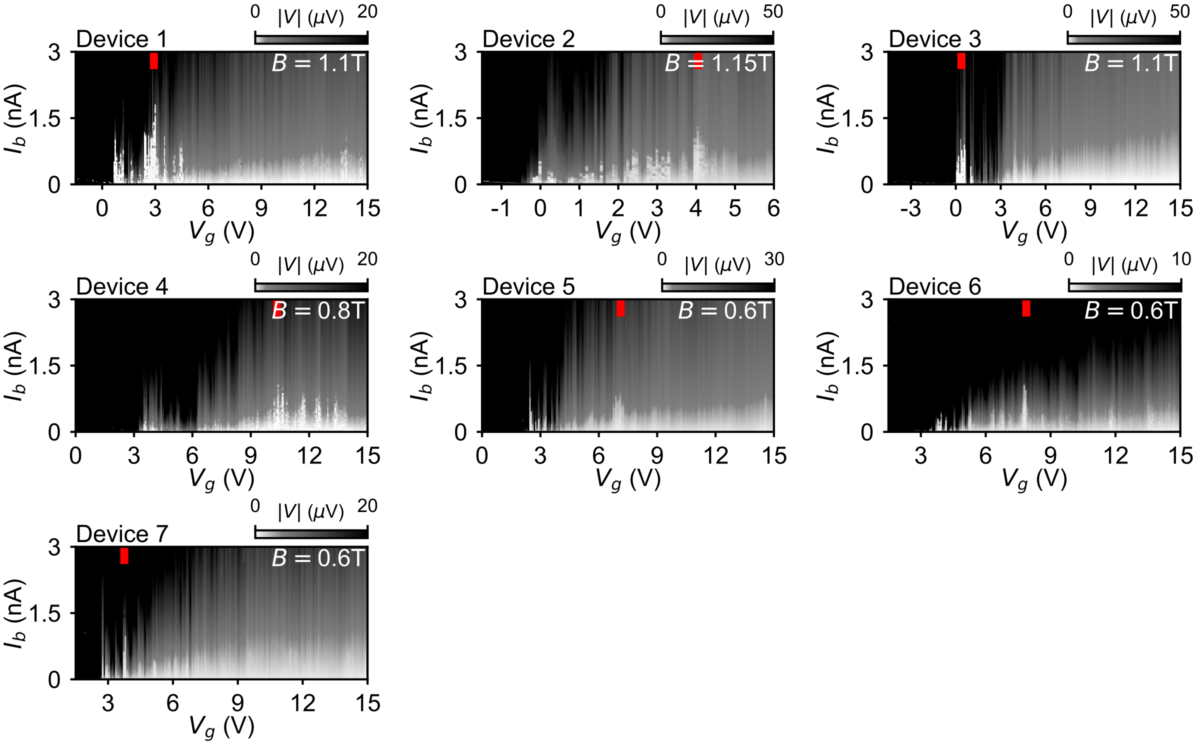

Figure S6: **Identifying the resilient gate settings** $V_{g,res}$: The back gate voltage V_g is swept at high parallel magnetic field for Device 1-7. The red markers denote the resilient gate settings $V_{g,res}$. V_g is set to these values for obtaining the magnetic field dependences shown in Fig. 3 and Fig. S7. The analogous measurements were not performed for Device 8-9.

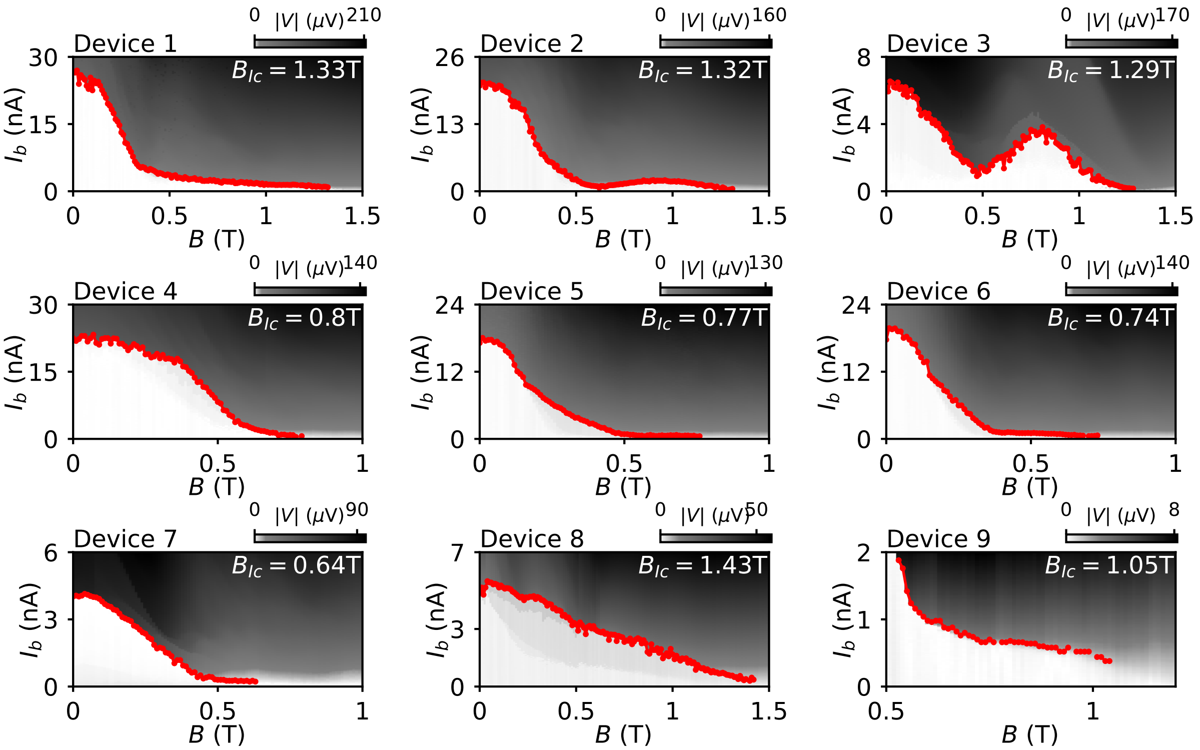

Figure S7: **Evolution of switching current in parallel magnetic field:** Dependence of the switching current I_{sw} (red) on the parallel magnetic field B for Device 1-9. The back gate is set at the resilient gate setting $V_g = V_{g,res}$ for Device 1-7 and at $V_g = 15\,\mathrm{V}$ for Device 8-9 (see the Data selection and reproducibility section). The corresponding extracted critical field B_c is shown as an inset. The gate settings for Device 1-7 are marked by the red markers in Fig. S6.

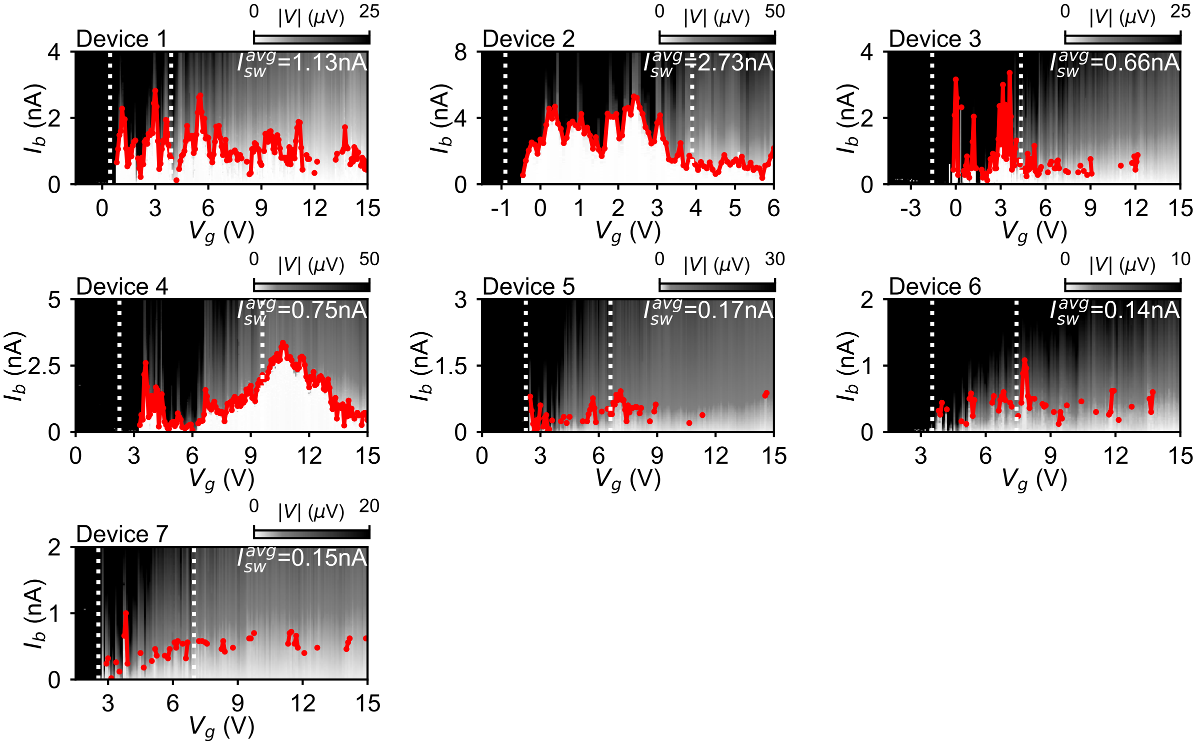

Figure S8: **Switching current at** $B = 0.6\,\mathrm{T}$ **parallel magnetic field:** Dependence of the extracted switching current I_{sw} (red) on the back gate voltage V_g at the parallel field $B = 0.6\,\mathrm{T}$ for Device 1-7. The white dotted vertical lines indicate the ranges of V_g over which the normal state conductance G_n at zero-field of the corresponding device increases from $0.1G_0$ to $2G_0$. The average switching currents over these intervals are shown as insets. The analogous measurement was not performed for Device 8-9.

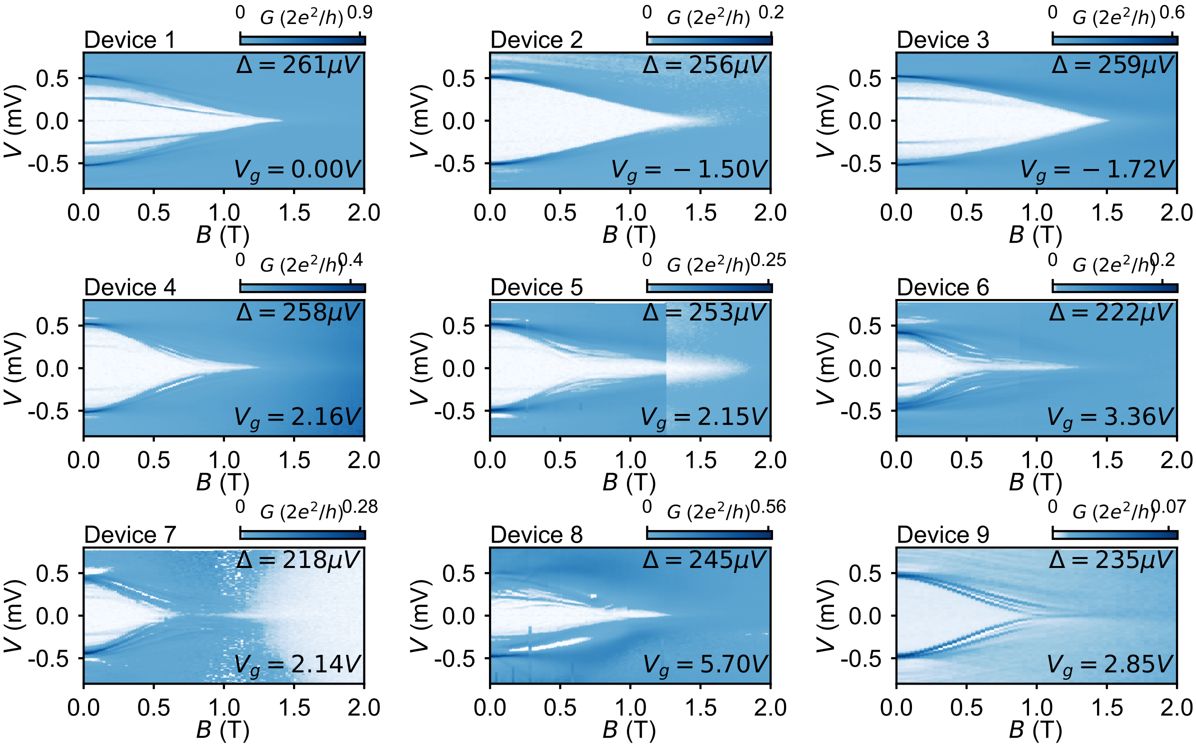

Figure S9: **Evolution of induced superconducting spectra in parallel magnetic field:** Dependence of the tunneling conductance G on the parallel magnetic field B for Device 1-9. Extracted induced superconducting gap at zero field Δ and the back gate voltage V_g at which the tunneling spectroscopy is measured for each device are shown as insets.

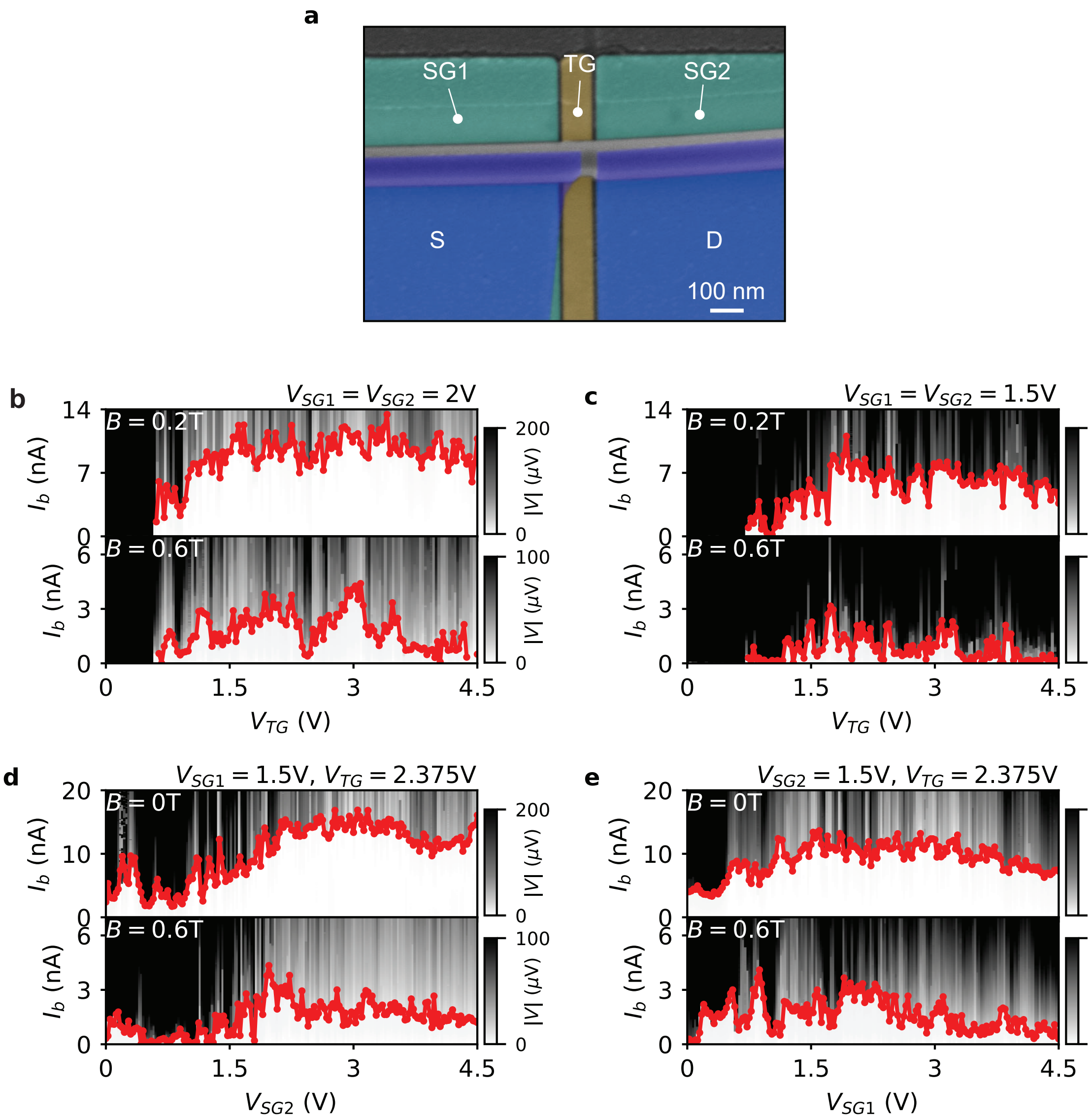

Figure S10: **Effects of local gates on supercurrent in the Josephson junction of Device 10**: Measurements were taken on a single arm of the SQUID, while the other arm was pinched-off. **(a)** False colour SEM image showing a 40 nm junction with the three local bottom gates: SG1, TG and SG2 (a zoom-in at the right junction of Fig. 5a; **(b)-(e)** Dependences of the extracted switching current I_{sw} (red) as a single local bottom gate voltage is swept while the other two local bottom gates and the parallel magnetic field are set as written in the corresponding insets.
